# The Complexity of Chinese Cereal Vinegar Flavor: A Compositional and Sensory Perspective

**DOI:** 10.3390/foods13050756

**Published:** 2024-02-29

**Authors:** Hong Zhu, Kehong Liang, Dazhou Zhu, Junmao Sun, Ju Qiu

**Affiliations:** 1 Institute of Food and Nutrition Development, Ministry of Agriculture and Rural Affairs, Beijing 100081, China; zhuhong@caas.cn (H.Z.); liangkehong@caas.cn (K.L.); zhudazhou@caas.cn (D.Z.); sunjunmao@caas.cn (J.S.); 2 Department of Nutrition and Health, China Agricultural University, No. 17 Tsinghua East Road, Haidian District, Beijing 100083, China

**Keywords:** Chinese cereal vinegar, aroma, brewing, lexicon, sensory wheel

## Abstract

With a millennium-long history, traditional Chinese cereal vinegar (CCV) is a significant part of China’s cultural heritage. The unique flavor of CCV is derived from the use of cereal and its bran as raw materials and solid-state fermentation as a brewing technique. This paper systemically summarized recent research progress on the aroma compounds in CCV, the biochemical generation of aroma compounds during the brewing process, and the association between sensory perception and the primary aroma compounds. Furthermore, a complete CCV lexicon and sensory wheel prototype were constructed. This study aims to lay a foundation for future CCV aroma research, quality improvement, and industrialization.

## 1. Introduction 

Traditional Chinese cereal vinegar (CCV) dates to the Zhou dynasty (1027–221 BCE) and has existed for thousands of years, with the first professional vinegar production workshop established in the Chunqiu Dynasty (770–476 BC) [1]. The classic solid-state fermentation technique used for traditional CCV has been perfected over thousands of years, propelled by intricate, natural microbial communities. Over 20 CCV types are commercially available in China.

The four most popular CCVs in China include Shanxi aged vinegar (SAV) (using a sorghum raw material and a *Daqu* starter), Zhenjiang aromatic vinegar (ZAV) (using sticky rice and a *Daqu* starter), Sichuan bran vinegar (SBV) (using wheat bran, various spices and herbs, and *Daqu*), and Yongchun *Monascus* vinegar (YMV) (using red yeast for saccharification, sticky rice, and liquid-state fermentation) [2]. Additionally, other CCVs, such as Beijing rice vinegar (BRV), Zhengrong rice vinegar (ZRV), and Zhejiang rose vinegar, are also popular. Unlike vinegars in other part of world, like Italian balsamic vinegar produced from grapes [3], Middle East vinegar from dates [4], and Philippines vinegar from coconut palm and sugar cane [5], the unique flavor of CCV can be attributed to the fermentation technique and raw materials used during production, including buckwheat, sorghum, corn, wheat, barley, and rice. CCVs are produced via solid-state fermentation, involving microbial proliferation on solid, moist substrates in the absence of free-flowing water, differing from the liquid fermentation technique used for Italian balsamic vinegar and sherry vinegar or slow traditional acetification processes (the Orléans or French method) for wine vinegar [6]. 

However, maintaining flavor quality, stability, and consistency between different CCV batches during traditional fermentation in open conditions is challenging due to a lack of systematic aroma compound assessment. Since aroma compounds crucially impact vinegar quality and sensory acceptability, their identification and quantitation in related products have attracted significant research attention over the past 10 years. The rapid advances in analytical methods and molecular sensory science have gradually started to reveal the chemical nature of CCV flavor and its formation mechanism. This study aims to promote the understanding of CCV aroma profiles, providing a theoretical foundation for qualitative and quantitative aroma compound research and quality control. 

## 2. Types of CCV 

### 2.1. Shanxi Aged Vinegar (SAV)

**Figure 1:**
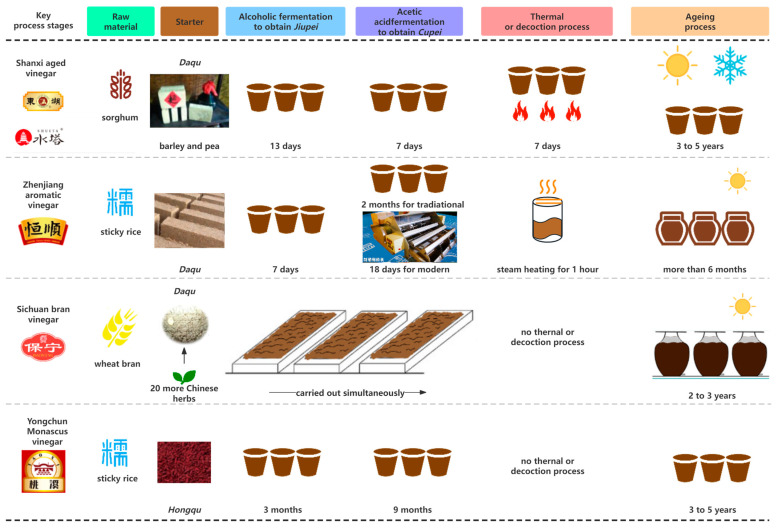
A flowchart of the CCV brewing process.

SAV, originating in the northern Chinese province of Shanxi, has been registered as a geographical indication (GI) product of China, forming part of its cultural heritage. Cultivated in the north of China, sorghum is the main ingredient in SAV, while other raw materials include rice hulls, millet bran, wheat bran, and peas. SAV is produced via solid-state fermentation in an open-style environment using various fungal and bacterial species. Traditional SAV fermentation involves several steps, including the preparation of the *Daqu*, saccharification of the starch, fermentation of the alcohol and acetic acids, thermal processing, and aging (Figure 1) [7,8]. *Daqu* is produced via spontaneous microorganism growth on peas and barley, accounting for approximately 60% of the raw material. The raw ingredients are fermented underground in a large jar for about 13 d, after which porosity is increased by mixing wheat bran and rice hulls with the alcoholic samples (*Jiupei* in Chinese) to enhance heat discharge and oxygen absorption. At the beginning of the 7 d acetic acid fermentation period, previous sample batch vinegar seeds (*Cupei*) are added to the jar. During the one-week thermal process, the *Cupei* is transferred to a jar, placed on a stove, and roasted. The aging process commonly lasts three to five years, involving water evaporation in the summer or freezing and collection in the winter. Although the SAV flavor, obtained via thermal processing, is typically sour and vinegary, it is also soft, fragrant, and mild.

### 2.2. Zhenjiang Aromatic Vinegar (ZAV)

ZAV, also known as Zhenjiang Xiang Cu in Chinese, was registered as both a GI product of China in 2001 and PGI of the EU in 2012 according to regulation (EU) No. 501/2012. ZAV has EU protection status under the PGI and PDO program, along with three types of balsamic vinegar products from Italy. ZAV is primarily manufactured using wheat bran, *Daqu* (consists of bacteria-, mold-, and yeast-containing fermented cereal), and sticky rice (mainly cultivated in southern China). Figure 1 shows a flowchart of the production process, which involves alcoholic liquid-state and acetic acid solid-state fermentation processes, leaching, decoction (liquid vinegar boiling), and aging. Acetic acid is fermented using either a traditional or industrial technique [9]. The traditional method is also known as the surface or slow technique since the acetic acid bacterial starter grows on the *Cupei* surface in a pottery jar and requires about two months of fermentation at 25 °C. The industrial, or modern, method involves submersion, where mechanical agitation is employed to provide the added acetic acid bacteria with the required oxygen to accelerate fermentation. This is a quick fermentation process that occurs in an oblong cement pool for about 20 d, involving lactic and acetic acid bacteria, mold, and yeast, to produce various metabolites. Furthermore, decoction and extended aging periods (several months or longer) may induce complex chemical reactions, such as esterification, amino acid and saccharide hydrolysis, and the Maillard reaction, in weakly acidic conditions, increasing the compositional complexity and exceptional flavor of ZAV.

### 2.3. Sichuan Bran Vinegar (SBV)

Sichuan Baoning vinegar, an SBV mainly produced in Baoning town, Sichuan province, primarily uses a wheat bran raw material, a *Daqu* saccharification starter, and various Chinese herbs. SBV is considered the sole medicinal Chinese vinegar due to the herbs used, such as *Villous amomum* fruit, *Eucommia* bark, and licorice, which promote fungal proliferation (i.e., *Rhizopus* spp.) in the *Daqu* during fermentation and contribute to the unique aroma profile [10,11]. The traditional brewing process includes *Daqu* production and fermentation. Solid-state SBV fermentation is a spontaneous, open process fueled by reproducible microbiota involving simultaneous acetic acid and alcohol fermentation and saccharification [12]. Similar to ZAV, this process can use both traditional and modern techniques [10,12]. Fermentation is followed by the aging process for two to three years in a pottery jar. During the day, the sealed vinegar is exposed to the sun while absorbing dew at night. Figure 1 shows the specific SBV production process. 

### 2.4. Yongchun Monascus Vinegar (YMV)

With a history of over 2000 years, YMV presents a unique flavor and bioactivity. YMV is recognized as a GI product produced in Yongchun County, Fujian Province. Unlike other traditional CCVs, YMV is produced via liquid fermentation using a glutinous rice raw material and a *Hongqu* fermentation starter, i.e., using glutinous rice to culture *Monascus* spp. [13]. YMV is placed in a pot or jar for an extended period to allow the development of the taste, flavor, and beneficial nutritional properties. 

### 2.5. Other CCV

Longmen rice vinegar, a famous brand of BRV, has a consumption history in the Beijing region of approximately 200 years. The BRV production process differs from other CCVs since fermentation directly follows rice grinding without thermal treatment (Figure 1) [2]. The raw materials of other CCVs are typically pretreated with steam. At the beginning of fermentation, the *Daqu* and yeast are incubated in a tank to promote alcohol formation. During acetic acid fermentation, rice husk and bran, as well as *Cupei* obtained from the previous batch of acetic acid fermentation, are introduced into the tank. The brewing process enters the aging phase, during which the flavor is enhanced. 

Traditional Chinese rose vinegar, which is highly popular in southern China due to its distinct color and flavor, is fermented via natural microorganisms falling into the fermentation container. The fermentation process consists of three stages: sticky rice starch degradation into sugars by mold, sugar conversion to ethanol by yeast, and acetic acid formation from ethanol via bacteria. Chinese rose vinegar tends to vary in different seasons since its quality is highly dependent on environmental factors due to the natural production process.

## 3. The Research Advancements on the CCV Flavor Components

Over the past 15 years, many qualitative and quantitative studies have investigated CCV aroma compounds by employing gas chromatography–mass spectrometry (GC–MS), two-dimensional gas chromatography–time-of-flight mass spectrometry (GC×GC–TOF-MS), gas chromatography–ion mobility spectrometry (GC–IMS), chromatography–olfactometry–mass spectrometry (GC–O–MS), and gas chromatography–olfactometry (GC–O). Advances in analytical methods have increased the number of identified CCV flavor components. Table 1 only lists some aroma-active components, given the significant number of identified volatile organic compounds. 

### 3.1. Shanxi Aged Vinegar (SAV) 

In 2011, Wang et al. analyzed the aroma profile of a Tartary buckwheat SAV using GC–O–MS [8], detecting 45 compounds, of which 24 and 15 were accurately and preliminarily determined, and 6 remained unidentified. In 2016, Zhu et al. created a technique to accurately determine the content of 23 volatile substances in SAV via SPME-GC–MS with both external and internal standards [18]. Of these, 19 were identified as aroma-active components by determining the odor activity values (OAVs), of which furfural, 3-methylbutanoic acid, acetoin, butanoic acid, trimethyl-oxazole, propanoic acid, and acetic acid presented the highest levels. In the same year, Liang et al. performed aroma extract dilution analysis by combining GC–MS, GC–O, and solvent-assisted flavor evaporation (SAFE) to compare the SAV aroma profiles before and after aging [14]. The results indicated that the SAV aroma composition before and after aging was almost the same, while the aroma compounds were altered. Specifically, the esters and pungent aroma were lost, while the Maillard reaction products, especially tetramethyl pyrazine, increased significantly. After SAV aging, the compounds presenting significant flavor dilution (FD) factors (>128) included 3-hydroxy-2-butanone, 2-ethyl-6-methylpyrazine, 2,3-dimethylpyrazine, furfural, 2-acetylpyrazine, dimethyl trisulfide, phenylacetaldehyde, 3-(methylthio)-propyl acetate, guaiacol, γ-nonalactone, 3-methylbutanoic acid, tetramethylpyrazine, 2,3-butanedione, vanillin, and 3-(methylthio)-propanal. 

### 3.2. Zhenjiang Aromatic Vinegar (ZAV) 

In 2012, Yu et al. used SPME-GC–MS [19] to identify 58 ZAV volatile compounds, including 13 acids, 9 alcohols, 5 aldehydes, 16 esters, 8 heterocycle compounds, and 4 ketones. In 2017, Zhou et al. characterized ZAV aroma compounds by combining GC × GC with GC–O and TOFMS [16]. A total of 360 substances were preliminarily determined according to the linear retention indices, mass spectra, and structured chromatograms, with ketones being the most abundant, followed by the ester, furan (and derivatives), aldehyde, and alcohol levels. The aroma-active elements were identified via comparison with the corresponding odors of the determined substances, and included 2-methyl-butanoic acid, phenethyl acetate, 3-methyl-butanoic acid, furfural, benzeneacetaldehyde, 3-methyl-butanal, 3-(methylthio)-propanal, trimethyl-pyrazine, acetic acid, dimethyl trisulfide, 2-methyl-butanal, octanal, 1-octen-3-one, and 2-methyl-propanal.

In 2019, Al-Dalali et al. used SPME-GS–MS and GC–O to compare traditional ZAV aroma profiles with modern ZAV during aging [9], identifying 53 volatile compounds, of which 43 were positively determined based on comparison with standard compounds. Furthermore, aroma compound differences were evident between the traditional and modern ZAV during aging. For example, isopentyl alcohol, 2-acetoxy-3-butanone, and 3-(methylthio)-propyl acetate were present in the traditional ZAV, while the modern vinegar contained octanoic acid, dimethyl trisulfide, propiophenone, and 2-ethyl-1-hexanol. The total number of volatile substances was higher in the modern ZAV than in the traditional samples. In 2020, Zhou et al. characterized ZAV aroma compounds using omission experiments, aroma recombination, GC–O–MS, and OAVs [20]. Sensory assessment showed that aged ZAV displayed a more intense buttery caramel odor and higher complexity than fresh ZAV. Here, 68 compounds were identified and evaluated, with the presence of sotolon detected for the first time in CCV. OAV calculation identified 27 odorants as crucial aged ZAV aroma compounds. Aroma recombination could effectively simulate the aged vinegar aroma profile, while omission experiments verified the role of the primary aroma compounds, including acetic acid, tetramethylpyrazine, 3-methylbutanoic acid, 2,4,5-trimethyloxazole, sotolon, 2-methylpropanal, and 2,3-butanedione.

### 3.3. Sichuan Bran Vinegar (SBV) 

In 2020, Al-Dalali et al. analyzed the key aroma substances and profiles in modern and traditional SBV via GC–O and SAFE-GC–MS [10]. They tentatively detected 99 volatile substances, of which 77 were then positively determined via comparison with standard compounds. Then, an aroma extract dilution assay (AEDA) was combined with GC–O to characterize 42 aroma-active compounds with FD factors (1 to 6561). Ten of these compounds were detected for the first time in CCV, while OAV calculation showed that 26 were key aroma compounds, with 3-oxobutan-2-yl acetate, acetic acid, furan-2-carbaldehyde, butyrolactone, and 2-hydroxy-3-butanone exhibiting the highest levels in both the traditional and modern SBV. Solution reconstitution showed that the SBV aroma profile could be closely simulated via aroma recombination in terms of fruitiness, roasted aroma, sweetness, woodiness, and spiciness while displaying slight differences in the herbal and nutty notes.

### 3.4. Yongchun Monascus Vinegar (YMV)

In 2019, Jiang et al. performed volatile metabolite analysis in the *Cupei* from different YMV brewing phases using SPME-GC–MS [13]. The study identified 60 volatile compounds, including 23 esters, 3 acids, 14 alcohols, 7 aldehydes, 3 alkanes, 4 ketones, 3 phenols, and 3 pyrazines. The alcohols and esters dominated during fermentation while acetoin, benzaldehyde, 2,3-butanedione, hexanoic acid, 1,3-butanediol, 2-methylbenzaldehyde, furfural, 2,3,5,6-tetramethylpyrazine, 4-ethylphenol, 2,3,5-trimethylpyrazine, benzeneacetaldehyde, and 2-methylpyrazine represented the most abundant volatile compounds during the late fermentation phase. A previous study used SPME-GC–MS and chemical isotope labeling liquid chromatography–mass spectrometry (CIL-LC–MS) to investigate the volatile and non-volatile metabolite changes during YMV aging [21], identifying 27 volatile compounds, including phenols, fatty acyls, organooxygen, and benzene and its substituted derivatives. Older YMVs displayed higher creosol, β-phenethyl-acetate, 4-ethyl-guaiacol, furfural, and acetylfuran ion intensities. Acetylfuran is considered a characteristic metabolite that differentiates aged vinegar, providing almond-like, sweet, balsamic, and caramel flavors. 

### 3.5. Other CCV 

In 2019, Al-Dalali et al. characterized Zhengrong rice vinegar (ZRV) and Longmen smoked vinegar (LSV) aroma profiles using SPME-GC–MS and GC–O [15], identifying 75 volatile compounds, of which 42 were confirmed via authentic substances. Higher ester and phenol levels were found in the ZRV, while the LSV contained more ketones, aldehydes, and pyrazines. In 2020, the same research team studied the aroma compounds of three kinds of Longmen rice vinegar using SPME-GC–MS and AEDA-GC–O [22], identifying 68 volatile compounds, of which 49 were verified via chemical standards. Higher aromatic hydrocarbon, acetal, sulfides, ester, alcohol, and acid concentrations were evident in the aromatic sweet rice vinegar sample due to the presence of additional sugar and solarization. The aromatic rice vinegar sample contained higher oxazole, lactone, phenol, ketone, pyrazine, and aldehyde levels, which could be attributed to added spices and solarization. In 2020, Zhao et al. examined the aroma profile of traditional Chinese rose vinegar using GC–MS–O and SPME-GC–MS [23], detecting 48 flavors and comparatively high acid and aldehyde concentrations. Furthermore, OAV calculation showed that aldehydes, such as dodecanal, heptanal, decanal, 3-methyl-butanal, and nonanal, likely contributed significantly to the aroma of this vinegar.

## 4. The Biochemical Generation of Key Aromas during the Brewing Process

Aroma compound variation was evident during different CCV fermentation stages. An abundance of heterocyclic compounds, alcohols, aldehydes, and esters was evident during the fermentation process, while thermal processing yielded ketones, aldehydes, pyrazines, and various other aroma substances, which were related to chemical processes like the Maillard reaction and hydrolysis. The aging process involves water evaporation, as well as chemical changes and reactions. The biochemical generation of the primary CCV aroma compounds are described in the following sections.

### 4.1. Raw Materials

While Europe traditionally uses fruit, mainly grapes, as raw materials for vinegar fermentation, Chinese vinegar is produced using cereals [24]. The cereal-based raw materials may be vital for CCV flavor descriptors, such as a licorice flavor. Aromatic aldehydes, including benzaldehyde, piperonal, vanillin, 3,5-dihydroxybenzaldehyde, and 5-methyl-2-phenyl-2-hexenal, can result from cereal-based raw materials, such as barley and sorghum [14]. 

### 4.2. Fermentation 

Significant heterocyclic compound, alcohol, ketone, aldehyde, and ester formation was evident during the SAV fermentation process. Nie et al. investigated the microbiota succession in traditional SAV during fermentation, indicating dramatic bacterial community compositional changes at different fermentation phases. The number of bacterial genera in the *Daqu* (relative abundance >0.1%) decreased from 17 to 2 at 12 d of alcohol fermentation. The 15 genera present at 1 d of the acetic acid fermentation decreased to 4 at 7 d, which included *Acetobacter* (50.9%), *Lactobacillus* (47.9%), *Komagataeibacter* (0.7%), and *Propionibacterium* (0.1%). The fungal community structure displayed more homogeneity, with *Saccharomycopsis* and *Saccharomyces* dominating during alcohol and acetic acid fermentation [25]. Re-inoculating the indigenous *P. manshurica* yeast strain into the *Daqu*-based fermentation increased the total ester, acetic acid, and ethanol levels in the SAV, producing a pleasant floral, fruity flavor and improving the aftertaste [26]. The activity of microorganisms can produce 3-hydroxy-2-butanone and 2,3-butanedione during the alcoholic fermentation of food products and beverages, such as wine.

Huang et al. demonstrated the effect of various starters on ZAV fermentation [27]. Acetic acid fermentation represented the primary phase for flavor compound formation during the brewing process. The acid content, including citric, lactic, and acetic acids, and volatile compounds, such as 2,3-butanedione and acetoin, continued to increase during the sealed fermentation phase. The bacterial and fungal community structures varied in the different fermentation stages. The dominant bacterial operational taxonomic units with average relative abundance values exceeding 10% in at least one fermentation phase included *Pseudomonas*, *Lactobacillus*, *Aeromonas*, *Acinetobacter*, *Acetobacter*, and *Acetilactobacillus*, while the most abundant fungal populations in each fermentation phase displayed obvious divergence, and included *Fusarium, Alternaria, Saccharomyces,* and *Wickerhamomyces*. 

SBV fermentation produces esters, acids, alcohols, carbonyls, heterocyclic compounds, and olefines [12]. The ester and acid contents were correlated with *Bacillus*, *Oceanobacillus*, *Virgibacillus*, *Paenibacillus*, *Trichosporon*, and *Rummeliibacillus*, while the olefin, carbonyl, and heterocyclic compound levels were significantly positively associated with *Acetobacter* (*p* < 0.05). Furthermore, the alcohol content displayed a positive correlation with *Hyphopichia*, *Monascus*, *Issatchenkia*, *Rummeliibacillus*, *Saccharopolyspora*, *Pichia*, *unclassified_o_Saccharomycetales*, *unclassified_f_Saccharomycetales_fam_Incertae_sedis*, *Aspergillus*, and *Pediococcus* (*p* < 0.05). 

Valine, lactic acid, esters, alcohols, alanine, and acetic acid represented the main aroma compounds during YMV fermentation, with *Yarrowia lipolytica*, *Sterigmatomyces halophilus*, *Saccharomycopsis fibuligera*, *Lactobacillus acetotolerans*, and *Komagataeibacter medellinensis* denoting the most abundant microorganisms [13].

### 4.3. The Thermal and Decoction Processes

The thermal and decoction processes are typical steps during the production of SAV and ZAV, respectively, providing the distinctive burnt, caramel-like aromas of these vinegars. Two important chemical reactions occur during thermal and decoction processing, namely hydrolysis and the Maillard reaction. First, the residual microorganism metabolites, hemicellulose, protein, and starch are gradually hydrolyzed to reducing sugars and amino acids in weakly acidic conditions, which are then transformed into aroma compounds via the Maillard reaction, including pyrazines, aldehydes, and ketones.

Pyrazines represent characteristic CCV aroma compounds, which are rarely present in European vinegar, like red wine or sherry vinegar. Pyrazines are commonly found in roasted food and beverages, such as coffee, contributing to the desirable roasted flavor [28,29]. They are likely products of the Maillard reaction via Strecker degradation. The SAV contained 2,3-dimethylpyrazine, tetramethylpyrazine, and trimethylpyrazine [8]. Tetramethylpyrazine was selected as a quality marker for SAV according to the “Product of geographical indication-Shanxi extra aged vinegar” Chinese national standard. Here, 19 pyrazines, mostly saturated alkyl pyrazines, were detected in the ZAV [16]. The Maillard reaction is likely responsible for oxazole production during heat treatment in the presence of high amino acid and sugar levels. 

Aldehydes, especially furfural and its derivatives, substantially impacted the ultimate sensory perception of CCV, contributing nutty, roasted, and almond flavors. Furfural presented roasted and caramel aromas resulting from Strecker degradation at high temperatures [30]. The Maillard reaction also facilitated the formation of the potent aroma compound 5-Methylfurfural, while a considerable number of furans were detected in the ZAV [16]. 

### 4.4. Aging Process 

Aging is crucial for enhancing the unique flavor of CCV. During SAV aging, the water content continued to decrease via evaporation and ice formation in the open jar, while the volatile levels also tended to decline. In addition to physical changes, various complex chemical reactions occurred. Liang et al. compared the aroma substances in SAV prior to and following the aging process [14], showing that the active odor compounds were mostly the same, while their quantities differed. The increase in aroma substances like dimethyl trisulfide, phenylacetaldehyde, and tetramethylpyrazine and the presence of pyrazine compounds indicated that the Maillard reaction continued during aging. This was reasonable since the SAV was in reducing sugars, amino acids, and proteins. The change in the traditional ZAV aroma profile during aging was also investigated [9]. The results showed that the total aldehyde, ester, alcohol, ketone, furan, and pyrazine concentrations decreased after aging, possibly due to chemical evaporation. Contrarily, the content of some volatile compounds increased, including 2-acetoxy-3-butanone, 2-phenethyl acetate, ethyl benzene acetate, ethyl acetate, and furfural. 

## 5. The Correlation between Sensory Perception and Aroma Substances

OAV calculation and AEDA are two widely used techniques for establishing a connection between aroma compounds and sensory quality analytical data. OAV is used to link the quantitative information obtained via chemical analysis to sensory perception, providing an effective method to compare sample aroma profiles [31]. In terms of AEDA, the FD factor of a compound can no longer be regarded as its maximum dilution time [14]. A higher FD factor indicates a more significant compound contribution to the overall aroma perception.

Acids are primarily associated with the tart, sour flavor perception of CCVs. In SAV, 3-methylbutanoic acid, acetic acid, and butanoic acid displayed the most significant FD factor values at log_2_ FD = 13, log_2_ FD = 10, and log_2_ FD = 6, respectively [14]. The furans in the CCV presented FD factors (log_2_ FD) between 1 and 4, with most producing sweet, caramel aromas. In the SAV, the furfural, furfuryl alcohol, and 5-methylfurfural exhibited the highest levels at 248.01 μg/L, 35.08 μg/L, 16.81 μg/L, respectively [14]. The oxazole content, typically presenting a nutty flavor, ranged from 59.72 μg/L to 330.3 μg/L in the BRV samples [22]. Pyrazines generally present nutty, earthy, roasted, and green aromas. The SAV displayed a high FD factor and elevated tetramethylpyrazine, 2,3-dimethylpyrazine, ethylpyrazine, and trimethylpyrazine concentrations [14]. The ketones contributed buttery, fruity, and floral odors. The highest 2,3-butanedione concentration (328.29 μg/L) was evident in the SAV, with a significant FD factor value of log_2_ FD = 13, followed by 3-hydroxy-2-butanone and *β*-damascenone. Aldehydes present sweet, floral, herbal, and green aromas. The log_2_ FD values of the aldehydes in the SAV ranged from 0 to 14 [14]. Responsible for sweet and chocolate flavors, vanillin displayed a significant FD factor value of log_2_ FD = 14 and the highest concentration at 20.29 μg/L, followed by the respective benzaldehyde, 5-methyl-2-phenyl-2-hexenal, and phenylacetaldehyde levels. Esters typically emit a fruity aroma and are considered crucial for vinegar flavor. The FD values of the 16 esters in the SAV ranged from 1 to 9 and included 10 aliphatic esters and 6 lactones (e.g., *γ*-decalactone and *γ*-hexalactone). Responsible for coconut and sweet flavors, *γ*-nonalactone was vital for the overall aroma of ZAV [16], SAV [14], and BRV [22]. The phenols, presenting herbal, spicy, woody aromas, were important contributors to CCV flavor, displaying low volatility values and weak water solubility, with log_2_ FD values between 0 and 9 in SAV. The guaiacol level was the highest at 11.34 μg/L with an FD value of log_2_ FD = 9, followed by 4-ethylguaiacol at log_2_ FD = 3 and 4-methylguaiacol at log_2_ FD = 3. 

## 6. The Sensory Perception of CCV

### 6.1. Lexicon

A shared lexicon is essential to effectively analyze the sensory perception of food. Lexicons are organized, established, and standardized vocabulary lists that can be used to accurately evaluate the sensory qualities of food or beverages, enhancing communication between researchers, product developers, and business partners. Lexicons are currently available for wine [32], beer [33], green tea [34], and more recently, coffee [35] and spices [36]. 

A lexicon differs significantly from other sensory evaluation tools in three key ways. The first involves description. A lexicon is a solely descriptive tool and does not allow for ranking quality, nor does it have categories for “good” and “bad” features. The second involves quantification. For instance, using a lexicon enables researchers to specify that a product has a blueberry flavor or an aroma intensity of 4 on a 15-point scoring scale, in addition to merely stating that it has a blueberry flavor or aroma. This significantly increases the accuracy of CCV difference comparisons. The third involves replicability. If trained sensory professionals utilize a lexicon properly, the same CCV assessed by two different individuals will yield the same intensity score for each attribute, regardless of their location, past taste experiences, cultural background, or any other distinction. For example, evaluators in Shanxi and Jiangsu will both perceive “blueberry, flavor: 4.” Moreover, a sensory lexicon is a dynamic resource that will be revised over time to incorporate new characteristics and references.

A lexicon with accurate definitions and references that can consistently distinguish and describe the sensory attributes will be helpful to producers, salespeople, researchers, and consumers, among other stakeholders in the CCV value chain. This study reviewed all the possible descriptors for the aroma and flavor of CCVs, irrespective of the area of origin, type, and raw material (Table 2). Trained panels of tasters developed a lexicon for describing CCVs, which defined and referenced 23 significant olfactory descriptors, namely chocolate, toasted, meat broth, licorice, walnut, yogurt, fruity, glue, honey, leather, caramel, burnt, flour, vanilla, spicy, smoked, green, bitter almond, mildew, rancid, boiled vegetable, butter, and woody flavors [37]. However, the judges in this study were all of Italian descent and were not very familiar with CCV. Another Chinese study selected ten olfactory terms for CCV aroma, namely sour, sweet, burnt, sauced, fruity, bran, incense, incense smoke, grain, alcoholic, and flowery [38]. A comprehensive, standard, and dynamic lexicon is necessary for characterizing the aroma and flavor of CCVs in both English and Chinese. 

### 6.2. Sensory Wheel

The sensory wheel is a useful visual aid for characterizing the flavor attributes of food products. It works by gathering, categorizing, arranging, and summarizing particular sensory attribute descriptors to represent the flavor characteristics of tested samples. The sensory wheel was created using lexicons and the findings of a professional sensory panel via a sensory evaluation [36]. Sensory wheels are more visual compared to lexicons. The sensory wheel helps producers better manage the quality of their products and provides the groundwork for future research and flavor discovery, which aids in both product development and improvement. The flavor wheel is currently employed in the tea [39], coffee [35], wine [40], and chocolate [41] industries, as well as various other fields.

Multivariate statistical methods are used to filter for precise and useful sensory descriptors. Next, to provide a summary, the descriptors are split into two or three levels. First-level terms fall under the macro category and are typically categorized based on how they are recognized (e.g., aroma, flavor, mouthfeel, and texture). First-level terms are used to refine and categorize second-level terms. Concrete object descriptors are referred to by the third-level terms. Finally, an image of a circular wheel represents the three levels of descriptors [42]. Multivariate statistical methods were utilized in the construction of the sensory wheel. Agglomerative hierarchical clustering (AHC), for instance, was utilized to identify the primary categories, subcategories, and tiers. Additionally, multidimensional scaling (MDS) was utilized to ascertain how these categories and subcategories were arranged within the wheel structure [35]. 

**Figure 2:**
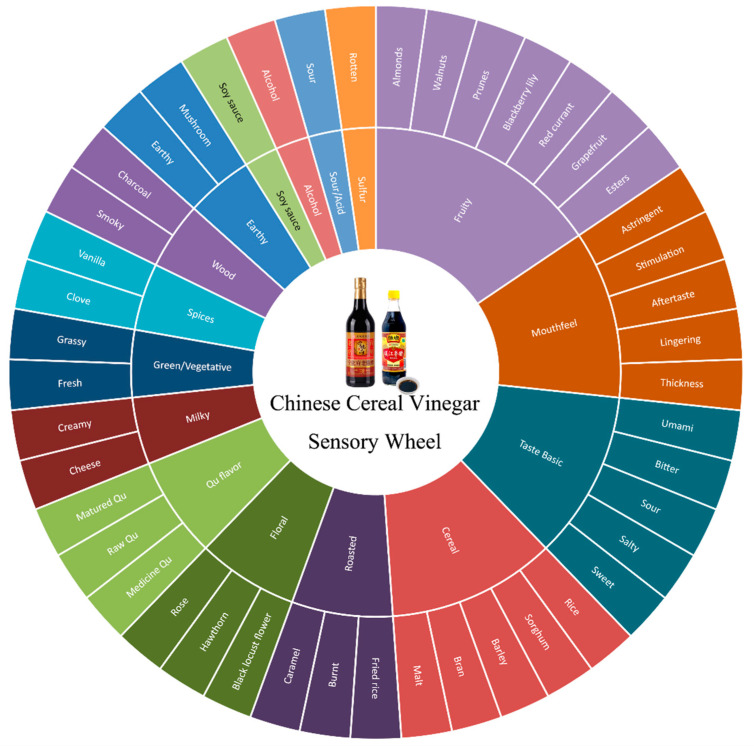
A sensory wheel of the CCV.

An initial version of the CCV sensory wheel was first built by Kong et al., who included a total of 45 sensory descriptors in 16 categories (Figure 2) [38]. The CCV sensory wheel is divided into several sections to help consumers visualize the different flavors, scents, and aromatic qualities found in most CCV products, including SAV, ZAV, and SBV. However, compared to other food products and beverages, like wine and coffee, more systematic research is required to construct a practical, accurate CCV sensory wheel. Innovative statistical and sensory techniques are necessary for constructing a flavor wheel from a defined lexicon. 

## 7. Conclusions and Future Trends

With a long history, traditional CCV derives its distinct flavor from two elements: solid-state fermentation, used in the brewing process; and cereal and bran as raw materials. However, traditional fermentation in non-sterile, open conditions makes it difficult to maintain consistent, stable flavor quality in different CCV batches. The traditional chemical composition and formation mechanism of CCV flavor is gradually revealed due to rapid advancements in analytical techniques and molecular sensory science. This study systemically reviewed the flavor compounds identified in CCV, aroma compound generation during the brewing process, and the association between sensory perception and key aroma substances. In addition, the CCV lexicon was compiled, and a CCV sensory wheel prototype was constructed. As a result, 101 volatile compounds including acids, pyrazines, furans, aldehydes, esters ketones, alcohols, phenols, were chosen as important aroma compounds of CCV according to the available literature. Primary strains in *Daqu* and Maillard reaction via Strecker degradation were considered to play a key role in the formation of aroma compounds. A useful sensory wheel was built, including 45 descriptors in 16 categories, for the standardization of the sensory analysis of CCV.

Three areas of research can be pursued in greater detail in the future. First, new analytical techniques can promote the identification of more trace but important aroma compounds. Second, artificial intelligence and machine learning can be employed for flavor quality control based on well-known flavor compound formation mechanisms. Finally, a dynamic, standard sensory wheel should be constructed, like the coffee flavor wheel by the SCAA. 

## Figures and Tables

**Table 1:** Aroma-active compounds identified in CCV.

Compound	Olfactory Description ^a^	CAS No.		Previously Reported in Vinegar				Identification Method ^b^
			SAV	ZAV	SBV	TMV	BRV	
Acids								
Acetic acid	Acid, vinegar	64-19-7	[8,14]	[9,15,16]	[10]		[15,17]	RI, MS, S, odor
Propanoic acid	Pungent, rancid, soy	79-09-4	[8]					RI, MS, S, odor
Isobutyric acid	Sour, acid	79-31-2		[9,16]				RI, MS, S, odor
Isovaleric acid	Sweat, rancid	503-74-2	[8,14]	[9,15,16]	[10]		[15,17]	RI, MS, S, odor
Pentanoic acid	Sweat	109-52-4	[8]					RI, MS, S, odor
Hexanoic acid	Sour	142-62-1	[8]	[9,15,16]	[10]		[15,17]	RI, MS, S, odor
Heptanoic acid	Sour	111-14-8			[10]			RI, MS, S, odor
Octanoic acid	Sweat, cheesy	124-07-2	[14]	[9]	[10]		[17]	RI, MS, S, odor
N-decanoic acid	Sweet, floral	334-48-5					[17]	RI, MS, S, odor
2-Methylbutanoic acid	Yogurt	116-53-0	[8]		[10]			RI, MS, S, odor
2-Methylpropanoic acid	Stinky, sour	6228-78-0			[10]			RI, MS, S, odor
Pyrazines								
Methyl pyrazine	Popcorn	109-08-0	[14]			[13]		RI, MS, S, odor
Ethyl pyrazine	Peanut butter, woody	13925-00-3	[14]					RI, MS, S, odor
2,3-Dimethyl pyrazine	Nutty, peanut butter, cocoa	5910-89-4	[8,14]				[15]	RI, MS, S, odor
2,5-Dimethylpyrazine	Cocoa, roasted nutty	123-32-0	[8]					RI, MS, S, odor
2,6-Dimethylpyrazine	Roasted nuts, cocoa, roast beef	108-50-9	[8]					RI, MS, S, odor
Trimethyl pyrazine	Roasted, potato, musty	14667-55-1	[8,14]			[13]		RI, MS, S, odor
Tetramethyl pyrazine	Cocoa, coffee, roasted	1124-11-4	[8,14]	[9,15,16]	[10]	[13]	[15,17]	RI, MS, S, odor
2-Ethyl-5-methylpyrazine	Fruity, sweet	13360-64-0	[8]					RI, MS, S, odor
3,5-Diethyl-2-methylpyrazine	Nutty	18138-05-1					[15]	RI, MS, S, odor
Furans								
Furfural	Almond, sweet	98-01-1	[8,14]	[9,15,16]		[13]	[15,17]	RI, MS, S, odor
5-Methylfurfural	Almond, caramel	620-02-0	[8,14]		[10]			RI, MS, S, odor
2-Pentylfuran	Green bean	377-69-3		[9,16]				RI, MS, S, odor
5-Hydroxymethylfurfural	Buttery	67-47-0			[10]			RI, MS, S, odor
5-Methyl-2-acetylfuran	Sour, musty	1193-79-9					[15]	RI, MS, S, odor
Furfuryl alcohol	Burnt	98-00-0	[14]		[10]			RI, MS, S, odor
Furan-2-ylmethyl acetate	Banana, sweet	623-17-6			[10]			RI, MS, S, odor
Aldehydes								
3-Methyl-butanal	Malty	590-86-3	[8]		[10]			RI, MS, S, odor
2-Methyl-2-butenal	Green, fruity	1115-11-3	[8]					RI, MS, S, odor
Benzaldehyde	Bitter almond, burnt sugar,	100-52-7	[8,14]	[9,15,16]		[13]	[15,17]	RI, MS, S, odor
Benzene acetaldehyde	Hawthorne, honey, sweet	122-78-1	[8,14]	[9,15,16]	[10]		[15]	RI, MS, S, odor
Heptanal	Fatty, citrus, rancid	111-71-7	[14]					RI, MS, S, odor
3-(Methylthio)-propionaldehyde	Cooked potato	3268-49-3	[14]					RI, MS, S, odor
2-Nonenal	Orris, fatty, cucumber	60784-31-8	[14]					RI, MS, S, odor
Vanillin	Vanilla	121-33-5	[14]		[10]			RI, MS, S, odor
Octanal	Citrus, fatty, green, oily, pungent	124-13-0				[13]	[17]	RI, MS, S, odor
Undecanal	Oily, pungent, sweet	112-44-7					[17]	RI, MS, S, odor
Nonanal	Fatty, floral, green, lemon	124-19-6		[15]			[15,17]	RI, MS, S, odor
Decanal	Floral, sweet	112-31-2		[15]			[15]	RI, MS, S, odor
Dodecaldehyde	Mud stinky	112-54-9					[17]	RI, MS, S, odor
2-Phenyl-2-butenal	Floral, honey	4411-89-6		[15]	[10]		[15]	RI, MS, S, odor
5-Pethyl-2-phenyl-2-hexenal	Bitter	21834-92-4			[10]			RI, MS, S, odor
Esters								
Ethyl acetate	Sweet	141-78-6	[8]	[9,15]		[13]	[15,17]	RI, MS, S, odor
Ethyl butyrate	Apple	105-54-4					[17]	RI, MS, S, odor
Ethyl valerate	Yeasty, fruity	539-82-2	[8]			[13]		RI, MS, S, odor
Ethyl hexanoate	Apple peel, fruity	123-66-0				[13]	[17]	RI, MS, S, odor
Ethyl caprylate	Fruity, fatty	106-32-1					[17]	RI, MS, S, odor
Phenethyl acetate	Rose, honey	103-45-7	[8]	[9,16]	[10]	[13]		RI, MS, S, odor
Isoamyl acetate	Banana	123-92-2				[13]	[17]	RI, MS, S, odor
Hexyl acetate	Fruity, herbal	142-92-7					[17]	RI, MS, S, odor
Ethyl myristate	Ether	124-06-1	[8]			[13]		RI, MS, S, odor
*γ*-Nonalactone	Coconut, peach	104-61-0	[14]	[9,15,16]	[10]		[15]	RI, MS, S, odor
Ethyl lactate	Fruity	97-64-3	[14]		[10]	[13]		RI, MS, S, odor
Ethyl l(−)-lactate	Sweet, fruity	687-47-8					[17]	RI, MS, S, odor
3-(Methylthio)propyl acetate	Herbal	16630-55-0	[14]					RI, MS, S, odor
Succinic acid, diethyl ester	Wine, fruity	123-25-1	[8,14]			[13]	[15]	RI, MS, S, odor
γ-Decalactone	Peach, fatty	706-14-9	[14]					RI, MS, S, odor
2-Methyl-1-propyl formate	Pear-like, sweet	542-55-2					[17]	RI, MS, S, odor
Ethyl-2-hydroxycaproate	Rubber smell	124439-28-7					[17]	RI, MS, S, odor
Isoamyl lactate	Almond, nutty	19329-89-6				[13]	[17]	RI, MS, S, odor
Ethyl decanoate	Grape	110-38-3				[13]	[17]	RI, MS, S, odor
Ethyl phenylacetate	Fruity, sweet	101-97-3		[15]		[13]	[15,17]	RI, MS, S, odor
Phenethyl acetate	Rose, honey, tobacco	103-45-7		[15]			[15,17]	RI, MS, S, odor
Ethyl hexadecanoate	Waxy	628-97-7					[17]	RI, MS, S, odor
Ethyl benzoate	Fruity, sweet	93-89-0		[15]			[15]	RI, MS, S, odor
Ketones								
2-Butanone	Ether	78-93-3	[8]				[17]	RI, MS, S, odor
2,3-Butanedione	Buttery	431-03-8	[8,14]			[13]	[17]	RI, MS, S, odor
2,3-Pentanedione	Creamy, buttery	600-14-6	[8]					RI, MS, S, odor
2-Heptanone	Soapy	110-43-0	[8]			[13]		RI, MS, S, odor
3-Hydroxy-2-butanone	Buttery	513-86-0	[8,14]	[15,16]	[10]	[13]	[15,17]	RI, MS, S, odor
Acetophenone	Musty, floral, almond	98-86-2	[8]					RI, MS, S, odor
3-Acetoxy-2-butanone	Smelly, sweet	4906-24-5		[15]			[15,17]	RI, MS, S, odor
Propiophenone	Pleasant honey scent	93-55-0					[17]	RI, MS, S, odor
Phenyl acetone	Fruity	103-79-7					[15]	RI, MS, S, odor
Jasmone	Geraniol, woody	488-10-8		[15]				RI, MS, S, odor
Alcohols								
Ethanol	Sweet	64-17-5					[17]	RI, MS, S, odor
1-Propanol	Alcohol, pungent	71-23-8					[17]	RI, MS, S, odor
1-Pentanol	Balsamic	71-41-0	[8]					RI, MS, S, odor
2,3-Butanediol	Fruity	513-85-9	[8]	[9]			[17]	RI, MS, S, odor
2-Methyl-1-propanol	Wine, solvent, bitter	78-83-1					[17]	RI, MS, S, odor
2-Phenylethyl alcohol	Honey, spicy, rose, lilac	60-12-8	[8,14]	[9,15,16]	[10]		[15,17]	RI, MS, S, odor
3-Methyl-1-butanol	Whiskey, malty, burnt	123-51-3					[17]	RI, MS, S, odor
Hexanol	Resin, floral, green	111-27-3				[13]	[17]	RI, MS, S, odor
1-Octen-3-ol	Mushroom	3391-86-4					[17]	RI, MS, S, odor
Heptanol	Chemical, green	111-70-6					[17]	RI, MS, S, odor
2-Ethyl-hexanol	Rose, green	104-76-7			[10]	[13]	[17]	RI, MS, S, odor
Nonanol	Fatty, green	143-08-8					[17]	RI, MS, S, odor
3-Methylthio-propanol	Sweet, potato	505-10-2			[10]		[17]	RI, MS, S, odor
Benzyl alcohol	Sweet, floral	100-51-6	[8]		[10]		[17]	RI, MS, S, odor
*β*-Ethylphenethyl alcohol	Rose, floral	2035-94-1				[13]	[17]	RI, MS, S, odor
Phenols								
Guaiacol	Smoky, sweet, medicinal	90-05-1	[14]		[10]		[17]	RI, MS, S, odor
4-Methylguaiacol	Phenolic	93-51-6	[14]	[15]	[10]		[15,17]	RI, MS, S, odor
4-Ethylguaiacol	Spicy, clove	2785-89-9	[14]	[15]	[10]	[13]		RI, MS, S, odor
2-Methoxy-5-methylphenol	Animal stinky, sour	1195-09-1					[17]	RI, MS, S, odor
*p*-Cresol	Pungent	106-44-5					[17]	RI, MS, S, odor
Others								
Dimethyl trisulfide	Sulfuric, fishy, cabbage	3658-80-8	[14]					RI, MS, S, odor
2-Acetylpyrrole	Nutty, walnut, bread	1072-83-9	[14]		[10]			RI, MS, S, odor
Styrene	Balsamic, gasoline	100-42-5	[8]					RI, MS, S, odor
Trimethyl oxazole	Sweet, green	20662-84-4	[8]					RI, MS, S, odor
Benzothiazole	Gasoline, rubbery	95-16-9	[8]		[10]			RI, MS, S, odor
Toluene	Paint	108-88-3					[17]	RI, MS, S, odor

^a^ Description from the following database: http://www.flavornet.org (accessed on 8 February 2024). ^b^ Each compound was identified based on the following: RI, retention indices; MS, mass spectrometry; S, standard compound injection; odor, the analyte odor descriptions were compared with their corresponding standards, as well as with the literature.

**Table 2:** The descriptive terms and their Chinese translations.

No.	Descriptors	Terms Used to Describe Sensorial Attributes	References
1	Walnut (坚果味)	Hazelnut	[37]
2	Licorice (甘草味)	Anise, mint, ginger, tarragon, dry orange peel, lemon, menthol	[37]
3	Toasted (烘焙味)	Bread crust, bread, alcohol, flour, yeast	[37]
4	Burnt (烧焦味)	Burnt bread	[37,38]
5	Caramel (焦糖味)	Balsamic, plum, dark beer, cooked must	[37]
6	Leather (皮革味)	Tannin, urine, farm, stable	[37]
7	Vanilla (香草味)	Christmas cake, cappuccino, chocolate hazelnut	[37]
8	Smoked (烟熏味)	Rhubarb, spices	[37,38]
9	Meat broth (肉汤味)	Cooked, fish, soy sauce	[37]
10	Bitter almond (苦杏仁味)	Coriander	[37]
11	Glue (胶水味)	Drugs, vinegar, acetic acid, camphor, chemical, incense, mothballs, paint	[37]
12	Chocolate (巧克力味)	Cocoa, coffee, hay, tobacco	[37]
13	Mildew (发霉味)	Rotten, moss	[37]
14	Honey (蜂蜜味)	Wax, candied fruit, raisins, molasses, wine	[37]
15	Rancid, sour (酸味)	Acid	[37,38]
16	Boiled vegetable (煮菜味)	Boiled spinach	[37]
17	Flour (面粉味)	Bread crust, bread, alcohol, toasted, yeast	[37]
18	Wood (木质味)	Sawdust, cork	[37]
19	Yogurt (酸奶味)	Creamy, pungent smell associated with sour milk	[37]
20	Fruit (水果味)	Typical smell of ripe fruit	[37,38]
21	Spice (香料味)	Chili-like, pepper-like, cinnamon-like	[37]
22	Green (青草香)	Fresh, plant-based material	[37]
23	Butter (黄油味)	Fatty, creamy smell of milk	[37]
24	Sauced (酱香)	Soy sauce	[38]
25	Bran incense (麸皮)	Wheat	[38]
26	Alcoholic aroma (酒香)	Chinese Baijiu	[38]
27	Floral (花香)	Floral aromatic	[38]

## Data Availability

The original contributions presented in the study are included in the article, further inquiries can be directed to the corresponding author.
